# Explorative Assessment of the Temperature–Mortality Association to Support Health-Based Heat-Warning Thresholds: A National Case-Crossover Study in Switzerland

**DOI:** 10.3390/ijerph20064958

**Published:** 2023-03-11

**Authors:** Martina S. Ragettli, Apolline Saucy, Benjamin Flückiger, Danielle Vienneau, Kees de Hoogh, Ana M. Vicedo-Cabrera, Christian Schindler, Martin Röösli

**Affiliations:** 1 Swiss Tropical and Public Health Institute (SwissTPH), 4123 Allschwil, Switzerland; 2 University of Basel, 4001 Basel, Switzerland; 3 Barcelona Institute for Global Health (ISGlobal), 08003 Barcelona, Spain; 4 Institute of Social and Preventive Medicine (ISPM), University of Bern, 3012 Bern, Switzerland; 5 Oeschger Center for Climate Change Research (OCCR), University of Bern, 3012 Bern, Switzerland

**Keywords:** case-crossover, temperature, heat waves, heat warnings, mortality, DLNM

## Abstract

Defining health-based thresholds for effective heat warnings is crucial for climate change adaptation strategies. Translating the non-linear function between heat and health effects into an effective threshold for heat warnings to protect the population is a challenge. We present a systematic analysis of heat indicators in relation to mortality. We applied distributed lag non-linear models in an individual-level case-crossover design to assess the effects of heat on mortality in Switzerland during the warm season from 2003 to 2016 for three temperature metrics (daily mean, maximum, and minimum temperature), and various threshold temperatures and heatwave definitions. Individual death records with information on residential address from the Swiss National Cohort were linked to high-resolution temperature estimates from 100 m resolution maps. Moderate (90th percentile) to extreme thresholds (99.5th percentile) of the three temperature metrics implied a significant increase in mortality (5 to 38%) in respect of the median warm-season temperature. Effects of the threshold temperatures on mortality were similar across the seven major regions in Switzerland. Heatwave duration did not modify the effect when considering delayed effects up to 7 days. This nationally representative study, accounting for small-scale exposure variability, suggests that the national heat-warning system should focus on heatwave intensity rather than duration. While a different heat-warning indicator may be appropriate in other countries, our evaluation framework is transferable to any country.

## 1. Introduction

Heat-related mortality is a global health problem [1,2]. Exposure to high ambient temperatures has been associated with all-cause mortality, with cardiovascular and respiratory diseases being identified as the main death causes [3]. A high vulnerability for heat-related mortality is especially observed among the elderly population and in people with pre-existing chronic diseases [4,5,6]. Several studies have reported a substantial burden of mortality attributable to non-optimal temperatures [7,8,9,10] and heatwaves [11,12,13,14]. For example, 345,000 heat-related deaths have been estimated globally among those older than 65 years in 2019. Compared to the 2000–2005 average, this constitutes an increase of 80.6%. With climate change and the ongoing increase in the frequency, intensity, and duration of heatwaves, the health impact of heat is likely to exacerbate [1,2,6].

Many countries have implemented heat-warning systems to protect population health during exceptionally warm weather periods. They are a common component of adaptation strategies and ensure a timely activation of prevention measures and emergency plans during heatwaves [15,16,17,18,19]. Some studies have demonstrated that such early warning systems in combination with public health actions are effective in preventing deaths during hot days, e.g., [20,21,22]. In some cities, however, the decline in heat–mortality association was insignificant [21,23]. 

For effective heat-warning systems, the determination of the meteorological parameters and thresholds triggering a warning should show a clear link with health effects. Threshold temperatures are ideally defined based on local characteristics considering both their frequency and associated health risks [16,17]. Although heat-related health effects are observed also during moderate warm temperatures, heat warnings are usually issued before extreme events to avoid too many heat advisory announcements. There is neither a standard definition of an extreme heat event nor a definition of a threshold to activating heat-health interventions [17,24]. It is well known from multi-city and multi-country studies that the effects of a specific temperature on health can vary between regions because of the populations’ adaptation to the regional climate and their sensitivity to heat [7,10,25]. Some warning systems such as in Germany and Spain also consider different thresholds at the beginning and the end of the summer to account for the short-term adaptation to heat in the course of a summer [17,26]. 

Assessments of the association between temperature and mortality provide insights into the health risk of temperatures beyond specific thresholds. Heat–mortality relationships are often quantified in time-series analysis, e.g., [10,27,28]. Such analyses investigate short-term relationships between the daily number of deaths of a given location and daily temperature data over a period of several years [29]. Weather data are mostly used from a single monitoring station per location or region; thus, spatial contrasts in exposure within the study area are ignored. Measurement campaigns have demonstrated a considerable variability in temperature within a city [30]. In Switzerland, for example, night-time temperatures were found up to 6–7 °C warmer in the city compared to the surrounding area [31]. To partially overcome this, some recent time series studies have used gridded temperature maps from climate models or interpolated weather data to derive population-weighted exposure estimates. Such estimates may better reflect the exposure that a population is experiencing on average, by giving more weight to zones with high population density [32]. Nonetheless, exposure misclassification cannot be ruled out, because the same exposure is assumed for the whole population. An alternative to time-series studies are case-crossover studies, for which individual-level death records can be used and, therefore, provide the possibility to accurately assign the exposure history of each death case [29,33]. 

In Switzerland, the climate-change-induced temperature rise and more frequent and severe heatwaves are of great concern. The annual number of days with daily maximum temperatures reaching 30 °C has increased substantially in recent years, and the average temperature increase since pre-industrial times (+2 °C) is nearly two times higher compared to the global mean [34]. In recent years, periods of hot weather have occurred more often, with particularly severe heatwaves in 2003, 2015, 2018, and 2019. The high ambient temperatures during the three warmest summers in 2003, 2015, and 2019 have caused an estimated 6.9%, 5.4%, and 3.5% excess mortality, respectively [14,35]. However, to date, studies investigating heat-related mortality risk in Switzerland have only been conducted for selected cities, based on aggregated health data or using exposure data from central monitoring stations of a city or from relatively coarse gridded maps [9,27,28]. 

The aim of this study was to investigate the non-linear relationship between temperature and mortality for daily mean (Tmean), daily maximum (Tmax), and daily minimum (Tmin) temperature using country-wide individual mortality data and high-resolution temperature estimates (100 × 100 m) in a case-crossover design with distributed lag non-linear models (DLNMs). From these analyses, we derived various potential heat-warning indicators based on threshold temperatures and days of duration taking into account regional variability and effect modification by demographics to support health-based heat warnings in Switzerland. Additionally, we tested the effect of heatwave duration on mortality for different threshold temperatures. 

## 2. Materials and Methods

### 2.1. Study Design

To assess the effect of heat on mortality in Switzerland, we used a case-crossover design where exposure at the day of death of a person is compared with corresponding exposures at selected control days. With this design, each case acts as his/her own control for individual time-invariant confounders [33]. In the time-stratified case-crossover design, the exposure to ambient temperature at the day of death (i.e., the ‘case’ event) is compared with the exposure at proximate days before or after the event (i.e., the ‘control’ events). As control days, we selected the same days of the week as the case day within the same month and year [36]. Each case day was, thus, matched with three to four control days. This enabled controlling for long-term and seasonal trends and for potential effects of the day of the week on mortality within the study area. 

We first estimated the relationship between temperature and mortality in Switzerland using all data and separately for seven major regions during the warm season (May to September) between 2003 and 2016. This allowed us to explore the effect of potential threshold temperatures for heat warnings. The regional analyses aimed to explore effect modification and determine whether acclimatization to regional climates requires different threshold temperatures for heat warnings. We examined the temperature–mortality association for the seven major regions defined by the Federal Statistical Office that represent similar environmental and population characteristics: North-West Switzerland, Espace Mittelland, Lake Geneva region, Zurich, Ticino, Central Switzerland, and Eastern Switzerland (Supplementary Material: Figure S1). In the second step, to further support health-based heat warnings, we assessed the effect of consecutive hot days on mortality to study how heatwave duration affects mortality. 

### 2.2. Mortality Data

We used individual death records from the Swiss National Cohort (SNC) over a 14-year period (2003–2016). The SNC is a long-term cohort based on the linkage of national census and mortality records for the whole Swiss population. The anonymized records included information on age and residential address [37]. We restricted the analyses to non-external deaths (International Classification of Diseases, 10th revision (ICD-10) codes A00-R99) among the permanent resident population occurring during the warm season (May to September). 

### 2.3. Temperature Data

We used 100 m resolution maps of Tmean, Tmax, and Tmin to extract daily ambient temperature levels at the home address for each of the case and control event days. The daily temperature grids cover the whole country, and were produced by the temperature model by Flückiger et al. [38]. This model predicts daily air temperatures separately for Tmean, Tmax, and Tmin at 2 m above ground level across Switzerland for the period from 2003 to 2018 leveraging satellite data, atmospheric re-analyses data, station-based temperature measurements, and land-use variables in a machine learning framework. The fine spatial resolution of 100 × 100 m allows for capturing intra-urban temperature variability, and represents a substantial improvement upon central monitors or coarser-resolution exposure maps (2 × 2 km) available for Switzerland [39]. Compared to similar temperature models developed for France or Northeastern USA [40,41], the individual models for the years 2003–2018 performed well in predicting temperature data with R^2^ ranging from 0.94 to 0.99 and RMSE from 1.05 to 1.86 °C. 

### 2.4. Statistical Analyses

#### 2.4.1. Temperature–Mortality Associations

We estimated the temperature–mortality associations in Switzerland separately for three temperature indicators (Tmean, Tmax, and Tmin). To produce the exposure–response functions, we ran conditional logistic regression models in a case-crossover study design with DLNMs [42]. The DLNM method allows for accounting for non-linear and delayed effects of temperature and relies on the definition of a cross-basis function. We modeled the exposure dimension of the cross-basis term with a quadratic B-spline with two internal knots placed at the 50th and 90th percentile of the warm-season temperature distribution for Tmean and Tmax, and with one internal knot placed at the 75th percentile for Tmin. The lag dimension was specified as a natural cubic spline with two internal knots placed at equally spaced values on the log scale. A maximum lag of seven days was used to capture lagged effects up to a week before death and to account for short-term harvesting. The applied model parameters were similar to previous studies investigating temperature–mortality associations during the warm season [9,27,43,44,45]. We selected the model parameters during preliminary analyses and validated them using Akaike’s criterion (AIC). All models also included an indicator variable for national public holidays.

#### 2.4.2. Threshold Temperatures

We present odds ratios (ORs) of mortality as the change in mortality risk from the median warm-season temperature to different potential threshold temperatures for heat warnings (90th, 92.5th, 95th, 98th, 99th, and 99.5th percentiles of the warm-season temperature distribution) to characterize the effect of heat intensity on mortality. These threshold temperatures are similar to previous heatwave studies [11,24,46,47] and represent the whole range of absolute temperature levels in terms of integers between the 90th and 99.5th percentile of Tmean, Tmax, and Tmin in our dataset (Table S1). We included the 99.5th percentile as a potential threshold because such extreme temperatures are likely to become more common in the study area [34]. To simplify the description and comparability of the results across the three temperature metrics, we refer to the thresholds as percentiles rather than absolute numbers. The median temperature was used as a reference as it approximately corresponds to the long-term optimum temperature in the study area at which temperature-related mortality risk is at a minimum during the warm season [27]. ORs are reported as cumulative risks over the whole lag period (lags of 0–7 days). 

#### 2.4.3. Evaluation of Regional Variability

We ran separate case-crossover models for each of the seven major regions to investigate whether the threshold temperatures corresponding to the 90th, 92.5th, 95th, 98th, 99th, and 99.5th percentile of the overall Tmean distribution across Switzerland result in different mortality risk estimates among the regions. While the health effect of a specific temperature may vary between regions because of different optimum temperatures, the shapes of the curves may differ. This could imply different effects of a certain temperature in respect of the reference temperature, even if the optimum temperature would be similar. Thus, we assessed the regional variability of threshold-related mortality effects based on two approaches. First, we computed the region-specific risk estimates by centering the exposure–response curves of each region on the overall median Tmean (17 °C) across the whole study area, ignoring regional differences of the optimum temperature. Knots of the exposure dimension of the DLNM cross-basis functions were placed at the same absolute temperatures as in the overall analysis for total Switzerland. Second, for each region, we investigated the effect of a given temperature threshold on mortality based on the deviation from the median of the region’s own temperature distribution. Following the approach of previous multi-country temperature-mortality assessments, e.g., [10], the placement of the knots in the exposure dimension of the cross-basis function was based on the region-specific temperature distribution (50th and 90th percentile). To additionally study whether the ORs of mortality associated with the exceedance of a given threshold varies between regions, we assessed the between-region heterogeneity (with the I^2^ statistics) using random-effects meta-analysis.

#### 2.4.4. Stratified Analyses by Age Group, Season, and Time Period

Additionally, we explored differences in effect estimates associated with the threshold temperatures by age group, season, and time period. For all temperature metrics, we conducted stratified analyses by two age groups (<75 years, 75+ years), for early (May to July) and late summer (August to September), and for two time periods (2003–2009 versus 2010–2016). The chosen time periods split the data series in two equal parts, with the more recent time period characterized by a higher health risk awareness of hot weather. For the analysis by time period, the model parameters of the DLNM were applied to the period-specific temperature distributions and the period-specific median temperature was used as the reference. As we were interested in the potential differences in effects of specific temperatures between the two time periods, we compared ORs of mortality associated with absolute temperature thresholds rather than with period-specific percentiles. As a sensitivity analysis, the year 2003 was excluded from the comparison, because it was an exceptional year in terms of heat-related mortality. 

#### 2.4.5. Effect of Heatwave Duration

To examine whether several consecutive days of high ambient temperatures have an additional effect on mortality versus a single hot day, we estimated the effect of different heatwave definitions by combining the various temperature thresholds explained in Section 2.4.2 with information on days of duration. As a first approach, we explored the effect of hot weather periods when Tmean reached at least the 90th, 92.5th, 95th, 98th, 99th, and 99.5th percentiles of the warm-season temperature distribution (i.e., May to September) during at least two, three, and five consecutive days. Thus, in total, 15 heatwave definitions with different intensities and durations were tested in relation to the mortality increase in Switzerland. A heatwave of, for example, three days occurred when a given threshold temperature was reached on a case or control event day and had been lasting for at least three days. We ran conditional logistic regression models for each heatwave definition by including an indicator variable for heatwave (yes/no) and an indicator variable for national holidays. 

In addition, as a second approach, similar to Gasparrini and Armstrong [46], we replaced the heatwave indicator by a numeric variable describing the number of consecutive days before and on the day of death during which Tmean has been above the threshold. For example, if someone died on the second day of any particular heatwave, the variable was set to two. The maximum length of heatwave duration was determined to be 10 days. We modeled the number of consecutive heatwaves days as penalized smoothing splines (p-splines) [48]. 

Models with both heatwave variables (heatwave indicator and numeric variable of heatwave duration) were run with and without the cross-basis function for Tmean to further explore the potential added effect of duration above the mortality impact of temperature and corresponding lagged effects up to seven days. 

We used R software (version 4.0.3) to conduct all data analyses, except for the meta-analysis, which we performed in STATA using the package metan [49]. The DLNM models were fitted in R with the *dlnm* package following the exposure history approach described by Gasparrini [50]. 

## 3. Results

In total, 300,295 deaths from natural causes were registered with complete address information in the SNC between the warm season of 2003 to 2016 (Table 1). The majority of the deaths (70%) occurred in people aged 75 years and older, and 51% of the study population were females. We observed no differences in temperature exposures by sex and age group. Across the whole study area, the median of estimated exposures to Tmean was 17 °C (range: 4–30 °C). The variability in exposure between regions was modest with the median Tmean ranging between 16 °C and 19 °C, representing the expected differences in regional climates. In the warmest region of Switzerland, Ticino, the median Tmean (19 °C), Tmax (25 °C), and Tmean (14 °C) were 2 °C to 3 °C higher compared to the respective overall median. The lowest median values of Tmean (16 °C) and Tmax (21 °C) were estimated for Eastern Switzerland, which is, on average, of higher altitude than the other regions. Overall, Tmean was highly correlated with Tmax (Pearson correlation coefficient: 0.97) and with Tmin (0.91). 

### 3.1. Exploration of Threshold Temperatures

**Figure 1:**
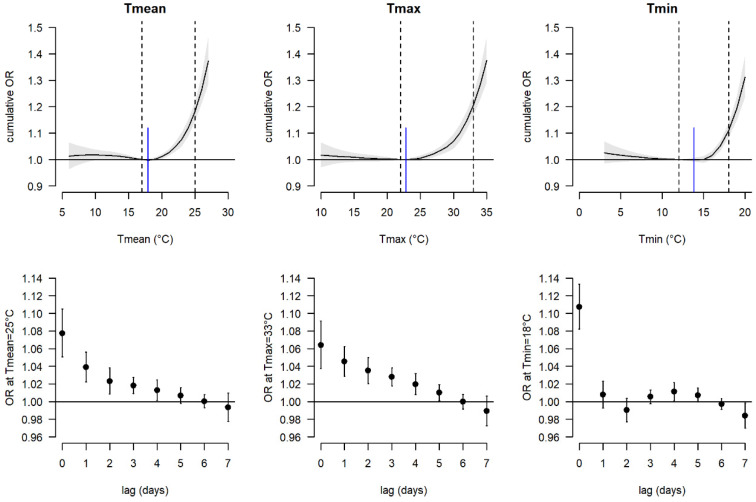
Odds ratios (ORs) of mortality associated with daily mean (Tmean), daily maximum (Tmax), and daily minimum (Tmin) temperature in Switzerland (May–September 2003–2016). Plots in the first row show the cumulative exposure–response association with the 95% confidence interval over one week (lags 0–7). Blue solid vertical lines are minimum mortality temperatures and dashed lines are the 50th (median) and 98th percentiles. Plots in the second row show the lag-specific ORs with 95% confidence intervals at the 98th percentile of the warm-season temperature distribution in respect of the median warm-season temperature.

Figure 1 presents the cumulative exposure–response functions for Tmean, Tmax, and Tmin, as well as the lag-specific ORs at the 98th percentile using the median temperature as a reference for the total study sample. Significant effects on mortality were observed for Tmean, Tmax, and Tmin at 20 °C, 26 °C, and 17 °C, respectively. Cumulative ORs of mortality for threshold temperatures corresponding to the 90th, 92.5th, 95th, 98th, and 99.5th percentile are provided in the Supplementary Material (Table S1). In general, a higher temperature threshold had higher effect estimates. When temperatures reached the 98th percentile of the warm-season Tmean (25°), Tmax (33 °C), and Tmin (18 °C), the mortality risk increased significantly by 18% (OR: 1.18 (95% confidence interval CI: 1.15; 1.22)), 21% (OR: 1.21 (1.17; 1.25)), and 11% (OR: 1.11 (1.08; 1.14)), respectively, over lags 0–7. Lower cumulative effect estimates for Tmin than for Tmax and Tmean partly arose by different lag patterns. While we observed increased ORs up to five days after the hot day (lag 0–5) for Tmax, warm nights only showed a strong immediate effect on mortality on the same day (lag0). Additionally, for Tmin, a significant increase in OR was only observed from 17 °C (corresponding to the 90th percentile) and higher. For Tmax and Tmean, mortality risk rose sharply after temperatures above the median value.

To assess whether the median temperature was an appropriate approximation of the minimum-mortality temperature (MMT) during the warm season, we also obtained the MMT from the temperature–mortality associations. The MMTs (indicated as blue lines in Figure 1) were similar to the median temperatures (left dashed lines in Figure 1). Thus, the mortality risks at the various threshold temperatures did not differ when using the MMT instead of the median temperature as reference. 

### 3.2. Analyses by Age Group, Season, and Time Period

For all inspected threshold temperatures above the 90th percentile of warm-season Tmean, Tmax, and Tmin, cumulative ORs were significantly higher in the older (75+ years) than in the younger age group (*p*-values ≤ 0.01) (Figure S2, Table S1). For people <75 years, significant effects were only observed for temperatures exceeding the 98th percentile (Tmean, Tmax) or 99th percentile (Tmin). The difference in effect among age groups was most evident for Tmin. The increase in mortality on lag 0 of a hot day with Tmin equal to 18 °C (98th percentile) was about two times higher in the population 75+ (OR = 1.13 (1.10; 1.16)) than in the younger age group (OR = 1.06 (1.02; 1.11) (Figure S2). 

**Figure 2:**
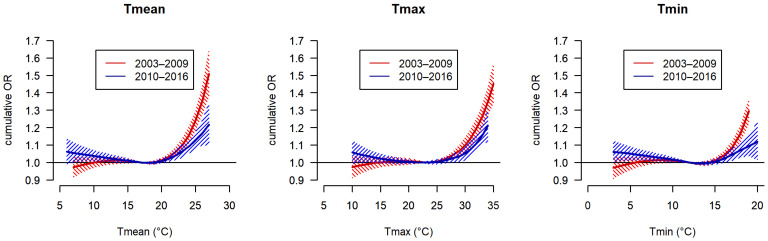
Odds ratios (ORs) of mortality associated with temperature in two time periods (2003–2009 and 2010–2016). ORs with 95% confidence intervals are shown for warm season (May–September) daily mean (Tmean), daily maximum (Tmax), and daily minimum (Tmin) temperature.

Stratified analyses by season showed higher associations with mortality in early than late summer (Table S2). The comparison of the two time periods (2003–2009 versus 2010–2016) revealed a decrease in heat sensitivity in the more recent time period (Figure 2), but temperature–mortality associations were significant for all threshold temperatures and temperature metrics in both periods (Table S3). For Tmean, the cumulative ORs of mortality at temperatures reaching 25 °C (98th percentile of warm-season temperature distribution in both periods) were significantly lower during 2010–2016 (OR = 1.13 (1.08; 1.18)) than during 2003–2009 (OR = 1.24 (1.19–1.30)). The difference remained statistically significant (*p*-value < 0.05) when excluding the year 2003 (Figure S3, Tables S4 and S5). 

### 3.3. Regional Variability

**Figure 3:**
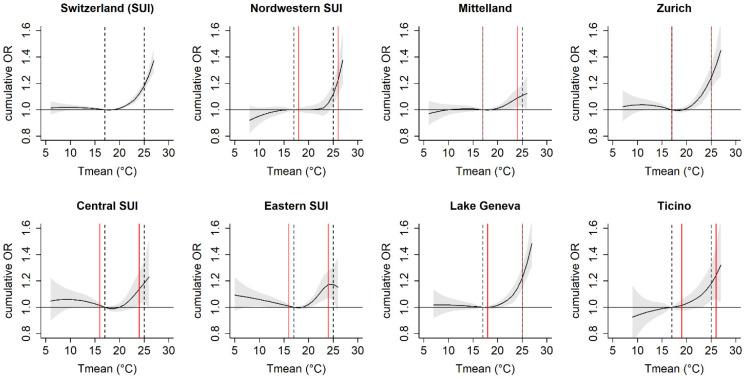
Region-specific odds ratios (ORs) of mortality associated with Tmean in Switzerland (May–September 2003 and 2016). Exposure–response associations are estimated based on the same (overall) model parameters and are reported for a cumulative 7-day lag versus the overall median of warm-season temperature across Switzerland. Vertical lines indicate the overall (grey dashed lines) and region-specific (red solid lines) 50th (median) and 98th percentiles.

**Figure 4:**
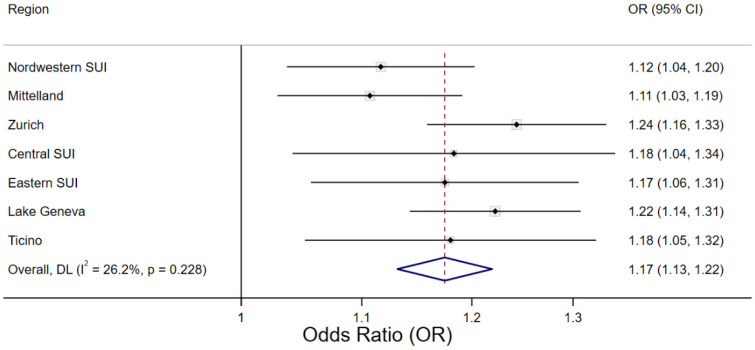
Forest plot of region-specific Odds Ratios (ORs) of mortality associated with Tmean exceeding 25 °C. The threshold of 25 °C corresponds to the 98th percentile of the daily mean temperature (Tmean) distribution during the warm season in Switzerland between 2003 and 2016. ORs are reported for a cumulative 7-day lag in respect of the overall median Tmean.

Region-specific associations between Tmean and mortality over the entire study period are shown in Figure 3, treating the overall median (17 °C) as the reference temperature. The shapes of the exposure–response curves of the seven regions were similar to the overall curve for Switzerland. We observed the highest cumulative ORs at the 98th percentile of warm-season Tmean (25 °C) for urban regions such as Zurich, the Lake Geneva region, and North-Western Switzerland. Heat-related ORs in the colder regions (Central and Eastern Switzerland) tended to be lower, but had a higher uncertainty in their estimates, especially for the very warm temperatures. However, as shown in Figure 4 for the threshold corresponding to the 98th percentile of warm-season Tmean, we observed no significant (*p* > 0.05) between-region heterogeneity for any of the thresholds. Thus, the region-specific OR of mortality associated with the exceedance of a particular threshold temperature did not statistically vary across regions. The forest plots of all Tmean thresholds are provided in the Supplementary Material (Figure S4). Estimating the threshold-related ORs of mortality of a given region using the region-specific median Tmean instead of the overall Tmean as the reference temperature (and, therefore, accounting for adaptation to the regional climate) did not change these results (Figure S5).

### 3.4. Effect of Heatwave Duration

Overall, we did not observe an added effect of heatwave duration on mortality when including a heatwave variable in the models assessing temperature–mortality associations using DLNMs. Neither the inclusion of a heatwave indicator (yes/no) nor the inclusion of a numeric variable of consecutive hot days had a significant impact on mortality risk (Table S6). 

**Figure 5:**
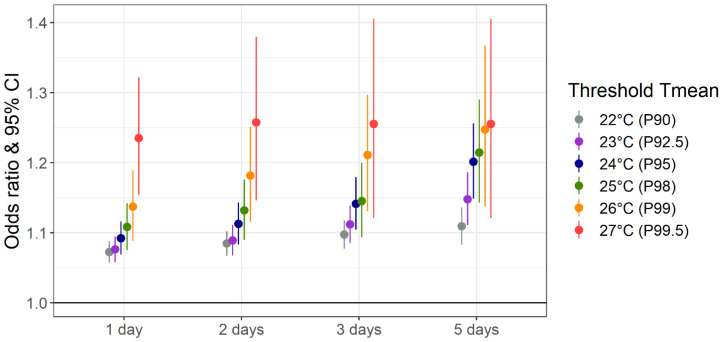
Effect of different heatwave definitions on mortality in Switzerland. Effects are shown for different daily mean temperature (Tmean) thresholds and days of duration, expressed as odds ratio (OR) comparing heatwave to non-heatwave days. P refers to the warm-season percentile of Tmean.

Figure 5 illustrates the effect of several consecutive hot days of different Tmean thresholds compared to non-heatwave days without accounting for delayed effects of hot days. ORs of mortality are shown for 15 heatwave indicators (yes/no), with the effect of one hot day for comparison. Longer durations tended to result in higher ORs. The influence of heatwave duration decreased with increasing temperature threshold and was virtually absent for the most extreme temperature threshold. While heatwaves with a threshold defined at the 90th percentile of warm-season Tmean (≥22 °C) over at least 2 to 5 days occurred in Switzerland every year between 2003 and 2016, extreme heatwaves with a Tmean threshold defined at the 99.5th percentile (≥27 °C) during at least five consecutive days were registered only for a few death cases (*n* = 235) during the hot summers 2003 and 2015 (Table S7). This explains the wide confidence intervals of the respective ORs. 

## 4. Discussion

We conducted a national case-crossover study to assess the temperature-mortality association for Tmean, Tmax, and Tmin during the warm season of 2003–2016 in Switzerland using individual-based death records and high-spatiotemporal-resolution (100 × 100 m; daily) temperature data. Temperatures exceeding the 90th, 92.5th, 95th, 98th, and 99th warm-season percentiles were significantly associated with substantial increases in mortality risk on the hot day itself and in the following week. We generally observed higher vulnerability in the elderly population and at the beginning of the summer. For the less vulnerable population <75 years old, no significant effect on mortality was observed for temperatures below the 98th percentile of Tmean and Tmax and below the 99th percentile of Tmin. Effects of the assessed threshold temperatures on mortality were similar across the seven major regions in Switzerland, suggesting that a common threshold temperature for heat warnings is valid for the whole country. The duration of a heatwave played a minor role in determining heatwave-related mortality risk. 

The shape of the association between heat and mortality observed in this study resembles that of a previous time-series study using data from eight cities in Switzerland between 1995 and 2013 [27]. After the exceptionally hot summer of 2003, several cantons have implemented public health measures to raise awareness on heat threats and to protect the health of vulnerable populations. Some cantons in the Lake Geneva region and the canton of Ticino have introduced heat-health actions plans (HHAPs) following the recommendations from the World Health Organization between 2004 and 2009 [51]. While the previous Swiss study found no significant decrease in health risk associated with high daily maximum apparent temperatures in the period after 2003 [27], we found significantly lower ORs of mortality associated with the Tmean threshold of 25 °C in the more recent decade (2010–2016) than between 2004 and 2009. This supports findings from studies in Italy, Spain, and Germany, which suggested that adaptive measures to the changing climate are effective [16]. It is possible that the public health measures introduced by cantonal HHAPs together with intensified media reports about heat-related health effects during heatwaves have helped to increase the heat-health risk perception and minimize the heat-related impact on mortality in Switzerland, especially in the warmest regions and among the elderly. Previous assessments of excess mortality during the warm summers 2015, 2018, and 2019 found a generally lower excess mortality in the Lake Geneva Region and in the Ticino than in other regions, despite more intense heatwaves [14,35]. However, how much prevention programs and heat alerts reduced the mortality risk during hot days is difficult to quantify as other improvements in public health, physiological adaptation, technological advances, and changes in the built environment may have helped to prevent heat-related deaths [18,52]. This includes, for example, policies aimed to improve the general health status of the population (e.g., smoking ban in public spaces), a wider application of air conditioning systems (mainly at workplaces and in public transport), and urban planning interventions toward greener cities. In addition, whether measures to increase the adaptive capacity of the population are still effective when heatwaves become both more frequent and intense and the population continues to age is unclear. It is likely that the health impact of heat will increase [2,6]. 

The mortality risk associated with different thresholds was similar among the seven regions. We did not observe a higher vulnerability to extreme temperatures in the colder regions (Central and Eastern Switzerland), which are expected to be least adapted to hot temperatures compared to the more urban areas (Zurich, Lake Geneva region, and North-Western Switzerland) and the South part of Switzerland (Ticino) where extreme temperatures are more frequent, also due to urban heat island effects. This may be surprising, as previous studies have shown that the vulnerability to heat varies spatially [10,25]. These studies generally covered cities from various countries, climates, and socioeconomic differentials, whereas in Switzerland, the climate varies over relatively small distances mainly due to the complex alpine topography. Most of the population, however, lives in urban and sub-urban areas (73%) and only a small percentage in mountainous areas [53]. While we cannot rule out that exposure–response functions vary by community, city, or canton, we found no evidence that the temperature threshold of heat warnings should be different between the seven major regions in Switzerland. A common heatwave definition in a small country such as Switzerland is advantageous, in that it simplifies communication of a heat alert. This is important for prevention given that many people commute between regions on a daily basis.

In agreement with previous studies, the intensity of a heatwave was more relevant for the mortality risk than its duration [11,46]. Similar to other studies, we observed delayed effects of up to four days of a hot day [9,45]. It has been suggested that these lagged effects are important to understand the health impact of heatwaves [11,54]. Long-lasting heatwaves may appear to have a higher impact on mortality than shorter periods of hot weather because of cumulating effects over several hot days. For instance, for a 3-day heatwave, the effects of the third day include the lagged effects of the first and second day. For the most extreme threshold (99.5th percentile of warm-season Tmean), an effect of duration was not observed. However, when we controlled for the lagged effects of Tmean (through DLNM), the effect of duration disappeared. Thus, the duration itself had no additional effect on the mortality risk of heatwaves. A similar result was also found in a large multi-country study by Guo et al. [11] that included data from 400 communities from 18 countries around the world and also considered lagged effects of heatwaves. DLNMs are, therefore, best suited to evaluate the combined effects of heat intensity and duration. The benefits of our simple models with threshold and duration indicators are that they directly provide an estimate of the mortality risk for certain heatwave warning definitions that may be considered to be implemented.

Consequently, for the development of a heat-warning system, the focus should be more on the intensity than on the duration of a heatwave. Even single hot days have an impact on mortality. However, it may not be advisable to issue high-temperature warnings that activate a range of public health measures for less than three forecasted consecutive hot days. This would not be cost-effective and would imply too frequent warnings and might cause the population to ignore them, thus rendering them ineffective [55]. Measures to be activated when issuing a heat warning include the dissemination of information on heat-related health risks and recommendations on how to protect health. Stakeholders in the health and social sector are advised to activate their emergency plans and other local interventions to protect the most vulnerable population [16,19]. 

Furthermore, while the duration may not be important for mortality, the impact of long-lasting heatwaves on other heat-related health aspects such as certain morbidity outcomes not assessed in this study may be greater [47,55,56,57]. Similarly, selecting the ‘best’ threshold used for heat advisories also implies a trade-off between associated health risks and the frequency of a given threshold temperature, both in the present and future. This may be an argument for using heat indicators, which are adapted to the regional climate, even if our study indicates that no major differences in exposure–response associations were observed across seven major regions. Especially in larger countries than Switzerland, regional heatwave definitions may be more relevant. It has to be emphasized that health risks associated with summer temperatures are observed even for temperatures below the evaluated thresholds (<90th percentile), especially in the elderly population. Thus, while heat warnings should be used to identify extreme and very extreme heatwaves, adaptation strategies are also needed to reduce the burden of moderate hot weather periods, which occur more often [10]. Further analyses are warranted to study threshold temperatures of specific risk populations such as people with specific diseases or sociodemographic characteristics to best protect them from heat exposure.

Recent research suggested that both high daytime and high nighttime temperatures are relevant for health. Although they strongly correlate, warm nights may make an important contribution to heat-related mortality especially among the elderly population because the body cannot recover sufficiently from the heat experienced during the day [27,43,44]. On the other hand, high daytime temperatures are also important because they mostly occur during afternoon hours when (outdoor) activities are carried out and they concern workers. Thus, to define heat-warning thresholds based on Tmean, which is an average over 24 h and, therefore, takes both nighttime and daytime hours into account, it may appropriately reflect the exposure of the whole day and also considers different heat vulnerabilities of the population [47]. The choice of Tmean for the determination of heat-warning thresholds may, therefore, be appropriate in Switzerland. However, as high Tmin usually occurs during the night when most people are at home, health risks during this time of the day may be mediated by urban heat islands and the insulation of the residential buildings. Recently, attempts to reduce heat exposure in buildings and urban city centers have become more important in Switzerland. The population with a high socio-economic status may profit more from such interventions than others [9]. Thus, health effects of high Tmin may become more variable than for Tmax. 

This is the first nation-wide assessment using individual-level data examining the temperature–mortality association for three temperature metrics for the whole country of Switzerland over a long time period (2003–2016). Our evaluation framework is transferable to any country to derive locally adapted heat-warning indicators. The strength of this study is that we used individual death records with information on residential address and temperature data of high spatial resolution (100 × 100 m) to assign exposure [38]. Exposure–response functions are, thus, representative for the whole country and take into account the small-scale variability in exposure within the study area. Our study likely provides unbiased and more precise effect estimates than most case-crossover and time-series studies that use health data with less detailed address information and exposure data of coarser spatial resolution. 

A limitation of this study is that we only assigned temperature levels at the residential addresses and ignored exposure while away from home. Thus, some exposure misclassification is to be expected, especially in the working population. Non-differential exposure misclassification could produce an underestimation of heat effects. However, the majority of the study population was ≥75 years old and was likely spending most of their time at home. Another limitation is that we did not consider other potential environmental risk factors such as air pollution. During periods of hot weather, ozone levels in particular tend to be high and may increase the health risk of heat. However, previous studies have found that there is an independent effect of temperature on mortality, and that the acute effect of ozone on mortality is relatively small [58,59]. Including air pollutants as potential effect modifiers in estimating the exposure–response function may complicate the interpretation of the direct heat effect. Additionally, for the public health intervention, it is of minor relevance whether deaths during a heatwave are related to heat or are the consequence of an indirect effect from elevated ozone concentrations. Similarly, relative humidity was not included, due to the lack of data of sufficient spatial resolution. Several countries use a heat index to describe the discomfort resulting from combined heat and high humidity. Previous studies in Switzerland and elsewhere showed, however, that temperature metrics considering humidity were highly correlated with Tmean and Tmax, and did not show significant differences in effect estimates [27,28,60]. Moreover, in recent years, there is a trend to favor heat-warning thresholds of dry temperature over more complicated heat indices because it is easier to derive and communicate them, and to study their frequency in climate scenarios (personal communication with MeteoSwiss).

## 5. Conclusions

This study presents a systematic analysis of heat indicators in relation to mortality across Switzerland over a period of 14 years. It adds to the existing body of evidence that heat is a relevant risk factor for mortality in Switzerland. It suggests that heat warnings in Switzerland using the same threshold temperature across the country are valid, because no major differences in effects were observed across seven major regions. As the intensity of a heatwave showed a more detrimental effect on mortality than the duration, the different levels of the heat-warning system to define the danger of a heatwave should rather be based on the intensity than on the duration of a heatwave.

## Figures and Tables

**Table 1:** Description of the number of deaths (by region and time period) and ambient daily mean temperature (Tmean), daily maximum temperature (Tmax), and daily minimum temperature (Tmin) on days of death and control event days during the warm season of 2003–2016.

	Deaths ^1^		Tmean (°C) ^2^				Tmax (°C)				Tmin (°C)			
	*n* (%)		p5	p50	p98	p99	p5	p50	p98	p99	p5	p50	p98	p99
Switzerland	300,295	(100)	10.1	17.0	25.0	25.9	13.8	22.4	32.5	33.6	6.0	12.3	18.5	19.1
Period 2003–2009 ^3^	149,989	(50)	10.2	17.1	24.9	25.7	14.0	22.5	32.4	33.7	5.9	12.4	18.3	18.9
Period 2010–2016 ^3^	150,306	(50)	10.0	16.9	25.2	26.0	13.6	22.3	32.6	33.6	6.1	12.2	18.6	19.3
Nordwestern Switzerland	41,583	(14)	11.1	17.6	25.5	26.5	14.8	23.2	33.5	34.6	6.7	12.8	18.6	19.1
Espace Mittelland	72,619	(24)	9.8	16.5	24.4	25.3	13.6	22.0	32.1	33.3	5.6	11.8	17.8	18.4
Zurich	50,462	(17)	10.4	17.1	25.2	26.0	13.8	22.4	32.8	33.9	6.4	12.6	18.4	18.9
Central Switzerland	25,785	(9)	9.6	16.5	24.3	25.1	13.1	21.6	31.7	32.7	5.8	12.0	17.9	18.4
Estern Switzerland	42,582	(14)	8.8	16.0	24.1	24.9	12.5	21.1	31.5	32.6	5.0	11.6	17.6	18.1
Lake Geneva region	53,419	(18)	10.7	17.6	25.5	26.5	14.8	23.1	32.7	33.9	6.5	12.8	19.1	19.8
Ticino	13,845	(5)	12.8	19.1	25.9	26.6	17.0	24.9	32.7	33.5	8.5	14.3	19.9	20.6

p5: 5th percentile; p50: median; p98: 98th percentile; p99: 99th percentile, ^1^ Includes natural deaths of permanent residents living in Switzerland; ^2^ Tmean refers to 24 h mean temperature; ^3^ Time periods split the entire study period in two equal parts, and the more recent time period is characterized by a higher heat risk awareness.

## Data Availability

The mortality data analyzed during the current study are available from the Swiss National Cohort Study Group but restrictions apply to the availability of these data, which were used under license for the current study, and so are not publicly available.

## Supplementary Materials

The following supporting information can be downloaded at: https://www.mdpi.com/article/10.3390/ijerph20064958/s1, Figure S1: Map of Switzerland showing the seven regions; Figure S2: ORs of mortality associated with Tmean, Tmax, and Tmin in Switzerland during the warm season (May–September) for two age categories; Figure S3: ORs of mortality with 95% confidence interval associated with Tmean, Tmax, and Tmin in Switzerland during the warm season (May–September) of two time periods (2004–2009 and 2010–2016); Figure S4: Forest plot of region-specific ORs of mortality associated with various Tmean thresholds in Switzerland; Figure S5: Estimates of cumulative ORs of mortality at various Tmean thresholds with respect to median warm-season Tmean in Switzerland and the seven regions during 2003–2016; Table S1: Cumulative ORs of mortality over 0–7 days for various temperature thresholds of Tmean, Tmax, and Tmin in relation to the respective median of the warm-season temperature distribution; Table S2: Cumulative ORs over 0–7 days associated with various temperatures of Tmean, Tmax, and Tmin in the overall study period (2003–2016, May to September) and during early (May to July) and late (August–September) summer; Table S3: Cumulative ORs over 0–7 days associated with various temperature thresholds of Tmean, Tmax, and Tmin in the overall study period (2003–2016) and during 2003–2009 and 2010–2016; Table S4: Cumulative ORs over 0–7 days associated with various temperatures of Tmean, Tmax, and Tmin during the time periods 2004–2009 and 2010–2016; Table S5: Description of the number of deaths and ambient Tmean, Tmax, and Tmin on days of death and control event days during the warm season by time period (2003–2009, 2004–2009, 2010–2016); Table S6: ORs of mortality associated with different threshold temperatures of Tmean in relation to the median of the warm-season temperature distribution. Table S7: Number of observations by heatwave definition based on Tmean during the study period (warm season 2003–2016) in Switzerland.
