# Supplementary material for: Explorative Assessment of the Temperature–Mortality Association to Support Health-Based Heat-Warning Thresholds: A National Case-Crossover Study in Switzerland
Source: Int J Environ Res Public Health. 2023 Mar 11;20(6):4958. doi: 10.3390/ijerph20064958 (PMC10049426; doi:10.3390/ijerph20064958)
Supplement: Supplementary file 1 [file ijerph-20-04958-s001.zip › ijerph-2194681-supplementary.pdf]

Supplementary Materials

Explorative assessment of the temperature–mortality association to support health-based heat-warning thresholds: A national case-crossover study in Switzerland

Martina S. Ragettli[1,2], Apolline Saucy[1,2,3], Benjamin Flückiger[1,2], Danielle Vienneau[1,2], Kees de Hoogh[1,2], Ana M. Vicedo-Cabrera[4,5], Christian Schindler[1,2], Martin Röösli[1,2]

[1] Swiss Tropical and Public Health Institute (SwissTPH), Allschwil, Switzerland
[2] University of Basel, Basel, Switzerland
[3] Barcelona Institute for Global Health (ISGlobal), Barcelona, Spain
[4] Institute of Social and Preventive Medicine (ISPM), University of Bern, Bern, Switzerland
[5] Oeschger Center for Climate Change Research (OCCR), University of Bern, Bern, Switzerland.

Figure S1. Map of Switzerland showing the seven regions.

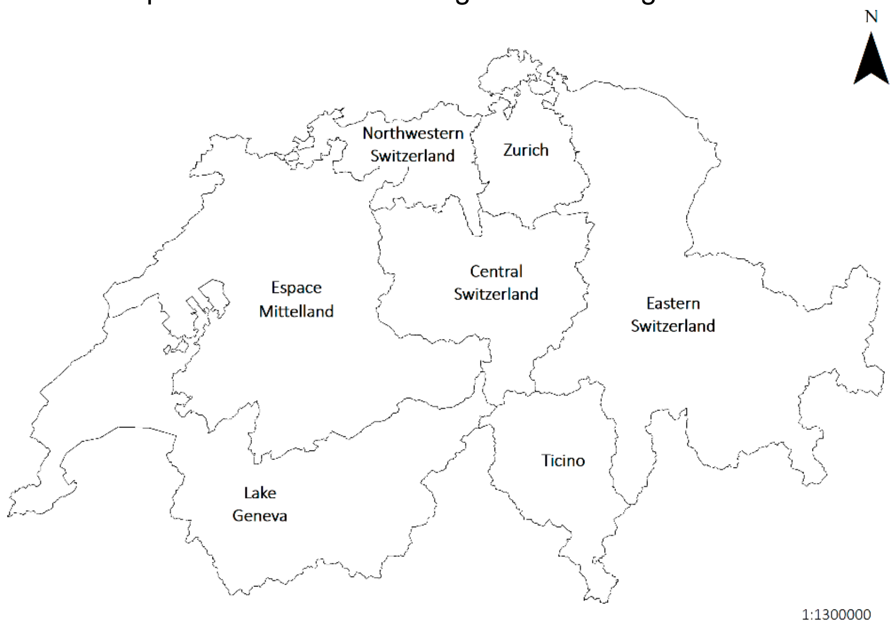

Boundaries: Swisstopo (2020), swissBOUNDARIES3D (accessed 03 February 2020).

Figure S2. Odds ratios (ORs) of mortality associated with daily mean (Tmean), daily maximum (Tmax), and daily minimum (Tmin) temperature in Switzerland during the warm season (May–September) for two age categories. Plots in the first row show the cumulative exposure–response association with 95% confidence interval over one week (lags 0-7). Plots in the second row show the lag-specific ORs with 95% confidence intervals at the 98th percentile of the warm-season temperature distribution in respect of the median warm-season temperature.

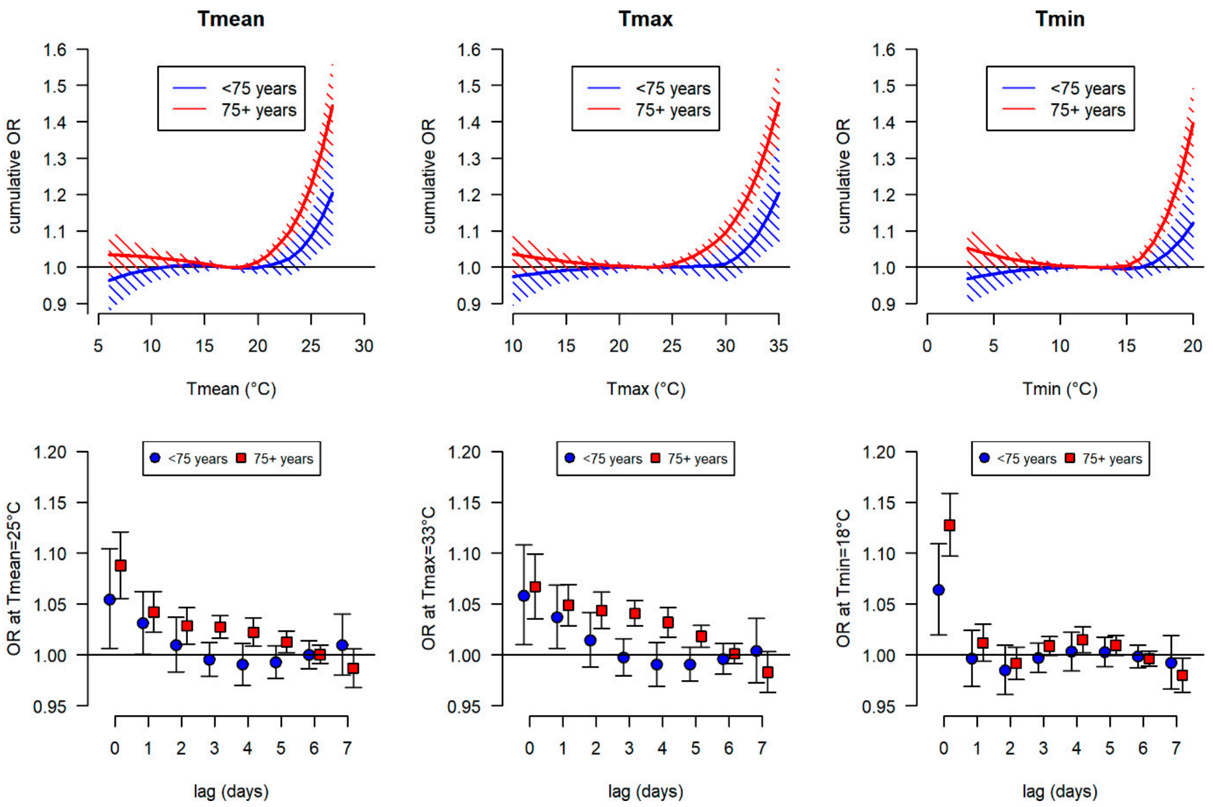

Figure S3. Odds ratios (ORs) of mortality with 95% confidence interval associated with daily mean (Tmean), daily maximum (Tmax), and daily minimum (Tmin) temperature in Switzerland during the warm season (May–September) of two time periods (2004-2009 and 2010-2016). The year 2003 was excluded from the analyses.

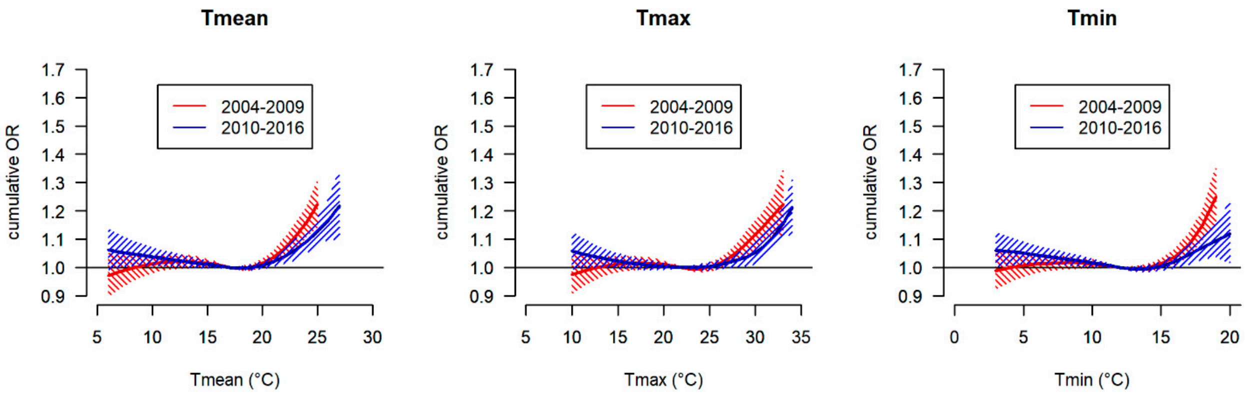

Figure S4: Forest plot of region-specific Odds Ratios (ORs) of mortality associated with various daily mean temperature (Tmean) thresholds in Switzerland. ORs are reported for a cumulative 7-day lag and are estimated based on overall (i.e., using the overall median Tmean as reference) model parameters. Threshold temperatures are shown as absolute values and as percentile (P) corresponding to the temperature distribution of the warm season in Switzerland between 2003 and 2016.

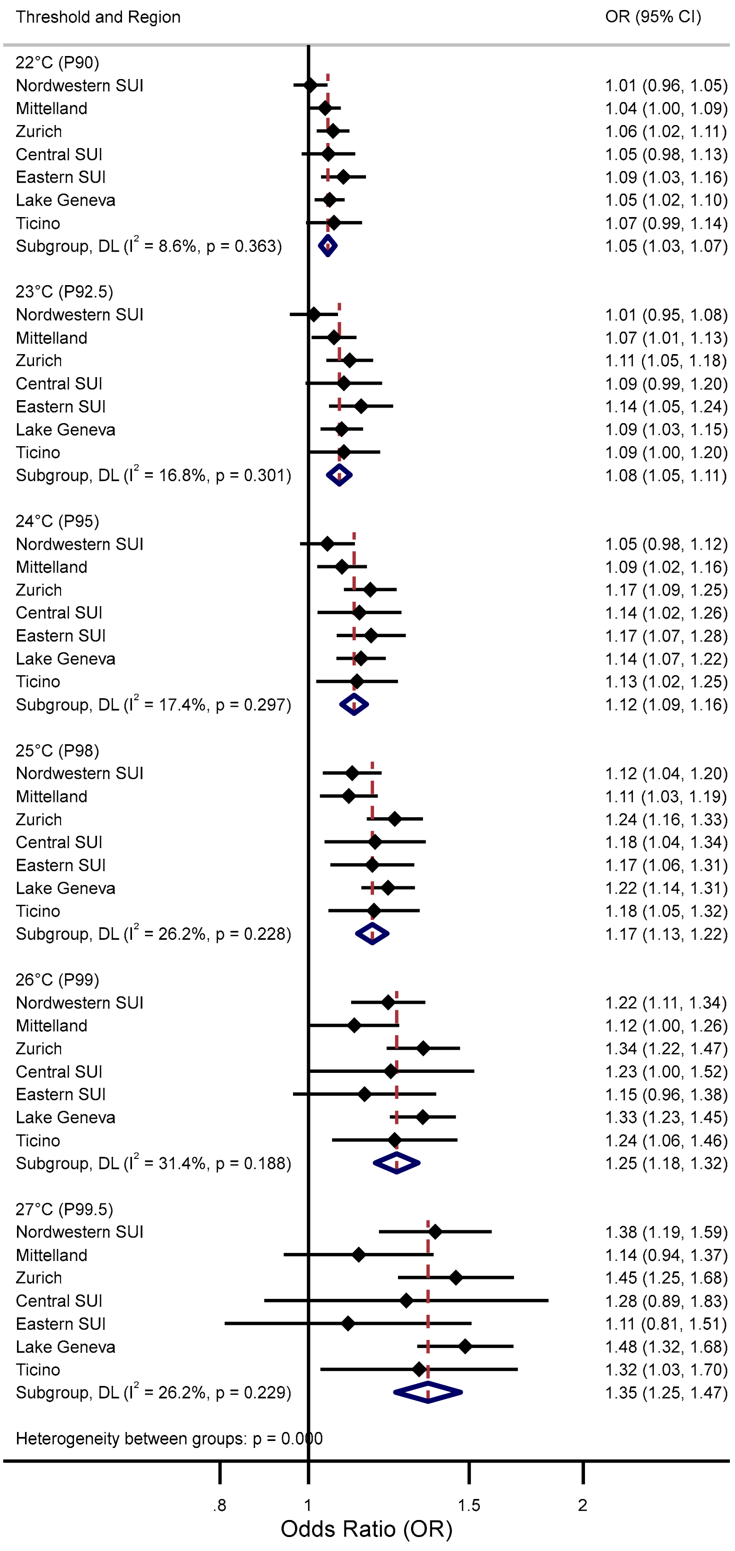

Figure S5: Estimates of cumulative odds ratios (ORs) of mortality at various daily mean temperature (Tmean) thresholds with respect to median warm-season Tmean in Switzerland and the seven regions during 2003-2016. ORs are reported for a cumulative 7-day lag and are estimated based on overall (i.e., using the overall median Tmean as reference) and region-specific model parameters (i.e., using the region-specific median Tmean as reference). Percentiles (P) of threshold temperatures refer to the Tmean distribution across total Switzerland.

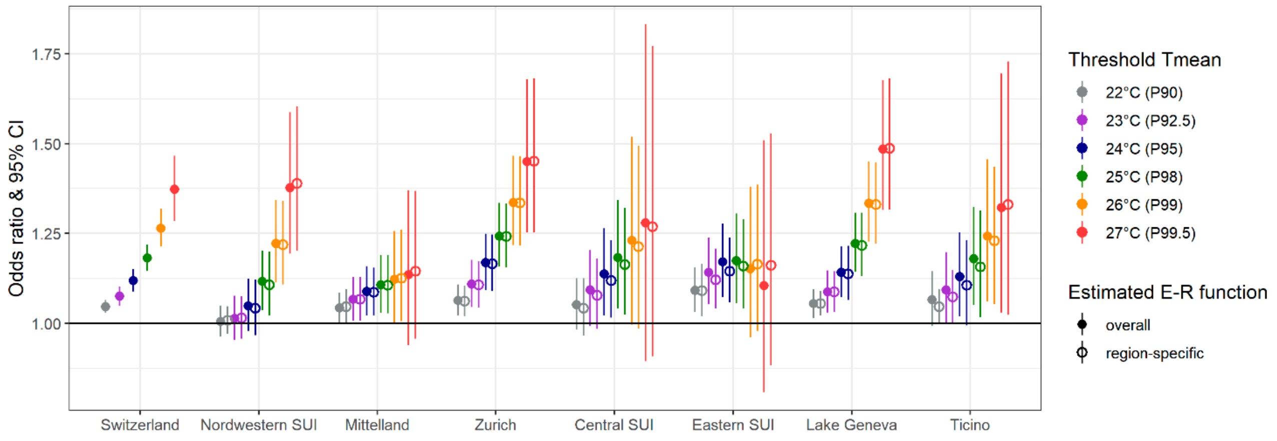

Table S1. Cumulative Odds Ratios of mortality over 0-7 days for various temperature (T) thresholds of daily mean (Tmean), daily maximum (Tmax), and daily minimum (Tmin) temperature in relation to the respective median of the warm-season temperature distribution. ORs are shown for the total population and two age groups.

	**Temperature threshold**	**Tmean**		**Tmax**		**Tmin**	
	Percentile (P)	rounded T (°C)	OR (95% CI)	rounded T (°C)	OR (95% CI)	rounded T (°C)	OR (95% CI)
total population	P90	22	1.05 (1.03; 1.07)	29	1.05 (1.03; 1.07)	17	1.05 (1.03; 1.07)
<75 years			1.01 (0.98; 1.04)		1.00 (0.97; 1.04)		1.01 (0.98; 1.05)
75+ years			1.06 (1.04; 1.08)		1.07 (1.04; 1.09)		1.07 (1.05; 1.09)
total population	P92.5	23	1.08 (1.05; 1.10)	30	1.07 (1.04; 1.10)	17*	1.05 (1.03; 1.07)
<75 years			1.02 (0.98; 1.07)		1.01 (0.96; 1.06)		1.01 (0.98; 1.05)
75+ years			1.10 (1.07; 1.13)		1.10 (1.06; 1.13)		1.07 (1.05; 1.09)
total population	P95	24	1.12 (1.09; 1.15)	31	1.10 (1.07; 1.13)	18	1.11 (1.08; 1.14)
<75 years			1.05 (0.99; 1.10)		1.03 (0.97; 1.08)		1.04 (0.99; 1.09)
75+ years			1.15 (1.11; 1.19)		1.14 (1.10; 1.17)		1.14 (1.11; 1.17)
total population	P98	25	1.18 (1.15; 1.22)	33	1.21 (1.17; 1.25)	18**	1.11 (1.08; 1.14)
<75 years			1.08 (1.02; 1.15)		1.09 (1.02; 1.16)		1.04 (0.99; 1.09)
75+ years			1.22 (1.18; 1.27)		1.26 (1.21; 1.31)		1.14 (1.11; 1.17)
total population	P99	26	1.26 (1.21; 1.32)	34	1.28 (1.23; 1.34)	19	1.20 (1.15; 1.24)
<75 years			1.14 (1.05; 1.23)		1.14 (1.05; 1.24)		1.07 (1.00; 1.15)
75+ years			1.32 (1.26; 1.39)		1.34 (1.28; 1.42)		1.25 (1.19; 1.31)
total population	P99.5	27	1.37 (1.29; 1.47)	35	1.38 (1.29; 1.47)	20	1.31 (1.24; 1.39)
<75 years			1.20 (1.07; 1.36)		1.20 (1.07; 1.35)		1.12 (1.00; 1.25)
75+ years			1.45 (1.34; 1.56)		1.45 (1.35; 1.56)		1.40 (1.30; 1.50)

*same rounded value as P90, **same rounded value as P95

Table S2. Cumulative Odds Ratios (ORs) over 0-7 days associated with various temperatures of daily mean (Tmean), daily maximum (Tmax), and daily minimum (Tmin) temperature in the overall study period (2003-2016, May to September) and during early (May to July) and late (August-September) summer in relation to the median of the warm-season temperature distribution (Tmean: 17 °C; Tmax: 22 °C; Tmin: 12 °C).

	**Temperature Threshold**	**Tmean**		**Tmax**		**Tmin**	
	Percentile (P)	rounded T (°C)	OR (95% CI)	rounded T (°C)	OR (95% CI)	rounded T (°C)	OR (95% CI)
overall	P90	22	1.05 (1.03; 1.07)	29	1.05 (1.03; 1.07)	17	1.05 (1.03; 1.07)
May-Jul			1.06 (1.04; 1.09)		1.06 (1.03; 1.09)		1.10 (1.07; 1.14)
Aug-Sep			1.02 (0.99; 1.05)		1.02 (0.99; 1.05)		1.02 (0.98; 1.06)
overall	P92.5	23	1.08 (1.05; 1.10)	30	1.07 (1.04; 1.10)	17	1.05 (1.03; 1.07)
May-Jul			1.10 (1.06; 1.13)		1.09 (1.05; 1.13)		1.10 (1.07; 1.14)
Aug-Sep			1.04 (1.00; 1.08)		1.03 (0.99; 1.08)		1.02 (0.98; 1.06)
overall	P95	24	1.12 (1.09; 1.15)	31	1.10 (1.07; 1.13)	18	1.11 (1.08; 1.14)
May-Jul			1.14 (1.10; 1.19)		1.12 (1.08; 1.17)		1.16 (1.12; 1.21)
Aug-Sep			1.07 (1.03; 1.12)		1.06 (1.01; 1.11)		1.06 (1.02; 1.10)
overall	P98	25	1.18 (1.15; 1.22)	33	1.21 (1.17; 1.25)	18	1.11 (1.08; 1.14)
May-Jul			1.21 (1.16; 1.26)		1.24 (1.18; 1.31)		1.16 (1.12; 1.21)
Aug-Sep			1.13 (1.08; 1.19)		1.16 (1.10; 1.22)		1.06 (1.02; 1.10)
overall	P99	26	1.26 (1.21; 1.32)	34	1.28 (1.23; 1.34)	19	1.20 (1.15; 1.24)
May-Jul			1.29 (1.21; 1.37)		1.33 (1.23; 1.44)		1.24 (1.17; 1.31)
Aug-Sep			1.22 (1.15; 1.29)		1.23 (1.16; 1.31)		1.13 (1.07; 1.19)
overall	P99.5	27	1.37 (1.29; 1.47)	35	1.38 (1.29; 1.47)	20	1.31 (1.24; 1.39)
May-Jul			1.39 (1.25; 1.53)		1.44 (1.28; 1.62)		1.34 (1.21; 1.48)
Aug-Sep			1.34 (1.22; 1.46)		1.33 (1.23; 1.43)		1.22 (1.12; 1.35)

Table S3. Cumulative Odds Ratios (ORs) over 0-7 days associated with various temperature thresholds of daily mean (Tmean), daily maximum (Tmax), and daily minimum (Tmin) temperature in the overall study period (2003-2016, n=300,295 deaths) and during 2003-2009 (n= 149,989 deaths) and 2010-2016 (n=150,306 deaths) in relation to the respective median of the warm-season temperature distribution (Tmean: 17 °C all periods; Tmax: 22 °C overall and 2010-2016, 23 °C 2003-2009; Tmin: 12 °C all periods). Significantly different ORs between time periods (2003-2009 versus 2010-2016) are shown in bold (Chi-square test, p-value <0.05). Period-specific percentiles corresponding to the given threshold temperatures are italicized.

Time period	Tmean				Tmax				Tmin			
	Threshold rounded T (°C)	Threshold Percentile (P)	OR (95% CI)	p-value	Threshold rounded T (°C)	Threshold Percentile (P)	OR (95% CI)	p-value	Threshold rounded T (°C)	Threshold Percentile (P)	OR (95% CI)	p-value
overall	22	P90	1.05 (1.03; 1.07)		29	P90	1.05 (1.03; 1.07)		17	P90	1.05 (1.03; 1.07)	
2003-2009		*P90*	1.06 (1.03; 1.08)			*P90*	1.07 (1.04; 1.1)			*P90*	1.08 (1.05; 1.12)	
2010-2016		*P90*	1.04 (1.01; 1.07)	0.304		*P90*	1.04 (1.01; 1.07)	0.159		*P90*	1.05 (1.01; 1.08)	0.127
overall	23	P92.5	1.08 (1.05; 1.10)		30	P92.5	1.07 (1.04; 1.10)		17	P92.5	1.05 (1.03; 1.07)	
2003-2009		*P92.5*	1.10 (1.06; 1.13)			*P92.5*	1.10 (1.06; 1.14)			*P92.5*	1.08 (1.05; 1.12)	
2010-2016		*P92.5*	1.06 (1.03; 1.10)	0.222		*P92.5*	1.05 (1.01; 1.09)	0.122		*P92.5*	1.05 (1.01; 1.08)	0.127
overall	24	P95	1.12 (1.09; 1.15)		31	P95	1.10 (1.07; 1.13)		18	P95	1.11 (1.08; 1.14)	
2003-2009		*P95*	**1.15 (1.11; 1.02)**			*P95*	1.14 (1.09; 1.19)			*P98*	**1.16 (1.12; 1.21)**	
2010-2016		*P95*	**1.09 (1.05; 1.14)**	**0.049**		*P95*	1.08 (1.03; 1.12)	0.062		*P95*	**1.07 (1.03; 1.11)**	**0.003**
overall	25	P98	1.18 (1.15; 1.22)		33	P98	1.21 (1.17; 1.25)		18	P98	1.11 (1.08; 1.14)	
2003-2009		*P98*	**1.24 (1.19; 1.30)**			*P98.5*	**1.26 (1.20; 1.33)**			*P98*	**1.16 (1.12; 1.21)**	
2010-2016		*P98*	**1.13 (1.08; 1.18)**	**0.002**		*P98*	**1.15 (1.10; 1.22)**	**0.014**		*P95*	**1.07 (1.03; 1.11)**	**0.003**
overall	26	P99	1.26 (1.21; 1.32)		34	P99	1.28 (1.23; 1.34)		19	P99	1.20 (1.15; 1.24)	
2003-2009		*P99*	**1.36 (1.28; 1.44)**			*P99*	**1.35 (1.27; 1.43)**			*P99*	**1.29 (1.23; 1.37)**	
2010-2016		*P99*	**1.17 (1.10; 1.24)**	**0.001**		*P99*	**1.21 (1.12; 1.31)**	**0.032**		*P99*	**1.10 (1.04; 1.16)**	**<0.001**
overall	27	P99.5	1.37 (1.29; 1.47)		35	P99.5	1.38 (1.29; 1.47)		20	P99.5	1.31 (1.24; 1.39)	
2003-2009		*P99.5*	**1.51 (1.38; 1.65)**			*P99.5*	1.45 (1.34; 1.57)			*>P99.5*	**1.49 (1.35; 1.64)**	
2010-2016		*P99.5*	**1.22 (1.10; 1.35)**	**0.002**		*P99*	1.28 (1.14; 1.45)	0.087		*P99.5*	**1.12 (1.01; 1.23)**	**<0.001**

Table S4. Cumulative Odds Ratios (ORs) over 0-7 days associated with various temperatures of daily mean (Tmean), daily maximum (Tmax), and daily minimum (Tmin) temperature during the time periods 2004-2009 and 2010-2016 in relation to the respective median of the warm-season temperature distribution (Tmean: 17 °C both periods; Tmax: 22 °C both periods; Tmin: 12 °C both periods). The year 2003 was excluded from the analyses. P-values represent the test for equality between the two time periods (Chi-square test, significance level <0.05).

Time period	Tmean				Tmax				Tmin			
	Threshold Rounded T (°C)	Percentile* (P)	OR (95% CI)	p-value	Threshold Rounded T (°C)	Percentile* (P)	OR (95% CI)	p-value	Threshold Rounded T (°C)	Percentile* (P)	OR (95% CI)	p-value
2004-2009	22	P90	1.07 (1.03; 1.10)		29	P92.5	1.08 (1.04; 1.13)		17	P92.5	1.08 (1.04; 1.12)	
2010-2016	22	P90	1.04 (1.01; 1.07)	0.194	29	P90	1.04 (1.01; 1.07)	0.088	17	P90	1.05 (1.01; 1.08)	0.214
2004-2009	23	P95	1.11 (1.06; 1.16)		30	P95	1.11 (1.07; 1.17)		17	P92.5	1.08 (1.04; 1.12)	
2010-2016	23	P92.5	1.06 (1.03; 1.10)	0.119	30	P92.5	1.05 (1.01; 1.09)	0.053	17	P90	1.05 (1.01; 1.08)	0.214
2004-2009	24	P98	1.16 (1.11; 1.21)		31	P98	1.15 (1.10; 1.20)		18	P98	1.15 (1.10; 1.20)	
2010-2016	24	P95	1.09 (1.05; 1.14)	0.044	31	P95	1.08 (1.03; 1.12)	0.037	18	P95	1.07 (1.03; 1.11)	0.020
2004-2009	25	P99	1.22 (1.14; 1.31)		33	P995	1.22 (1.12; 1.34)		18	P99	1.15 (1.10; 1.20)	
2010-2016	25	P98	1.13 (1.08; 1.18)	0.045	33	P98	1.15 (1.10; 1.22)	0.286	18	P98	1.07 (1.03; 1.11)	0.020
2004-2009	26	**			34	**			19	P995	1.25 (1.15; 1.36)	
2010-2016	26	P99	1.17 (1.10; 1.24)		34	P99	1.21 (1.12; 1.31)		19	P99	1.10 (1.04; 1.16)	0.008

*period-specific percentile

**corresponds to a temperature value above the 99.5th percentile

Table S5: Description of the number of deaths and ambient daily mean temperature (Tmean), daily maximum temperature (Tmax), and daily minimum temperature (Tmin) on days of death and control event days during the warm season by time period (2003-2009, 2004-2009, 2010-2016).

	Deaths*	Tmean (°C)				Tmax (°C)				Tmin (°C)			
	n (%)	p5	p50	p98	p99	p5	p50	p98	p99	p5	p50	p98	p99
Period 2003-2016	300’295 (100)	10.1	17.0	25.0	25.9	13.8	22.4	32.5	33.6	6.0	12.3	18.4	19.1
Period 2003-2009**	149’989 (50)	10.2	17.1	24.9	25.7	14.0	22.5	32.4	33.7	5.9	12.4	18.3	18.9
Period 2004-2009	127’308 (42)	10.1	16.8	24.0	24.7	13.8	22.2	31.2	32.1	5.9	12.2	17.9	18.4
Period 2010-2016**	150’306 (50)	10.0	16.9	25.2	26.0	13.6	22.3	32.6	33.6	6.1	12.2	18.6	19.3

P5: 5th percentile; p50: median; p98: 98th percentile; p99: 99th percentile

*includes natural deaths of permanent residents living in Switzerland; **time periods split the entire study period in two equal parts and the more recent time period is characterized by a higher heat risk awareness.

Table S6. Odds Ratios (ORs) of mortality associated with different threshold temperatures of daily mean temperature (Tmean) in relation to the median of the warm-season temperature distribution (17 °C). Results over the total lag-period (0-7 days) are shown with and without including a heatwave variable (heatwave indicator yes/no, numeric variable of consecutive days Tmean has been above the threshold on the day of death).

Temperature (°C)	Percentiles (P)	Tmean	Tmean & heatwave indicator (yes/no)		Tmean & numeric variable of consecutive heatwave days on case event (dur)
		Lag0-7	Lag0-7 + 3d HW	Lag0-7 + 5d HW	Lag0-7 + dur*
		OR (95%CI)	OR (95%CI)	OR (95%CI)	OR (95%CI)
22 vs 17	P90 vs P50	1.05 (1.03;1.07)	1.04 (1.02;1.06)	1.05 (1.03;1.06)	1.05 (1.02;1.07)
23 vs 17	P92.5 vs P50	1.08 (1.05;1.10)	1.07 (1.05;1.10)	1.07 (1.05;1.10)	1.09 (1.06;1.12)
24 vs 17	P95 vs P50	1.12 (1.09;1.15)	1.12 (1.09;1.15)	1.12 (1.09;1.15)	1.13 (1.09;1.16)
25 vs 17	P98 vs P50	1.18 (1.15;1.22)	1.19 (1.15;1.23)	1.18 (1.14;1.22)	1.20 (1.15;1.24)
26 vs 17	P99 vs P50	1.26 (1.21;1.32)	1.26 (1.20;1.32)	1.27 (1.21;1.32)	1.26 (1.19;1.33)
27 vs 17	P99.5 vs P50	1.37 (1.29;1.47)	1.36 (1.27;1.46)	1.35 (1.26;1.45)	1.30 (1.20;1.41)

*modeled as p-spline

Table S7. Number of observations by heatwave definition based on daily mean temperature (Tmean) during the study period (warm season 2003-2016) in Switzerland.

Def	Heatwave variable	Heatwave definition (Tmean threshold and duration)	case event days (day of death)	case and control event days	Days of duration Mean (range)*	years of occurrence
1	HW indicator (yes/no)	22°C (P90) with ≥2-d duration	27'665	116'886		2003-2016
2	HW indicator (yes/no)	22°C (P90) with ≥3-d duration	20'792	86'979		2003-2016
3	HW indicator (yes/no)	22°C (P90) with ≥5-d duration	12'295	50'960		2003-2016
4	HW indicator (yes/no)	23°C (P92.5) with ≥2-d duration	16'099	67'195		2003-2016
5	HW indicator (yes/no)	23°C (P92.5) with ≥3-d duration	11'122	45'648		2003-2016
6	HW indicator (yes/no)	23°C (P92.5) with ≥5-d duration	5'905	23'741		2003-2016
7	HW indicator (yes/no)	24°C (P95) with ≥2-d duration	8'565	34'875		2003-2016
8	HW indicator (yes/no)	24°C (P95) with ≥3-d duration	5'639	22'542		2003-2016
9	HW indicator (yes/no)	24°C (P95) with ≥5-d duration	2'973	11'442		2003, 2005-2016
10	HW indicator (yes/no)	25°C (P98) with ≥2-d duration	4'198	16'756		2003-2016
11	HW indicator (yes/no)	25°C (P98) with ≥3-d duration	2'760	10'911		2003, 2005-2016
12	HW indicator (yes/no)	25°C (P98) with ≥5-d duration	1'546	5'860		2003, 2006, 2009, 2010-2013, 2015
13	HW indicator (yes/no)	26°C (P99) with ≥2-d duration	1'811	6'988		2003, 2005, 2006, 2009-2016
14	HW indicator (yes/no)	26°C (P99) with ≥3-d duration	1'240	4'659		2003, 2006, 2010-2015
15	HW indicator (yes/no)	26°C (P98) with ≥5-d duration	643	2'400		2003, 2006, 2012, 2013, 2015
16	HW indicator (yes/no)	27°C (P99.5) with ≥2-d duration	689	2'522		2003, 2006, 2013, 2015
17	HW indicator (yes/no)	27°C (P99.5) with ≥3-d duration	445	1'612		2003, 2006, 2015
18	HW indicator (yes/no)	27°C (P98.5) with ≥5-d duration	235	790		2003, 2015
19	Numeric variable (duration)	nr of consecutive days with ≥22°C	37'633	160'661	4.2 (1-10)	2003-2016
20	Numeric variable (duration)	nr of consecutive days with ≥23°C	23'461	99'264	3.8 (1-10)	2003-2016
21	Numeric variable (duration)	nr of consecutive days with ≥24°C	13'280	55'248	3.0 (1-10)	2003-2016
22	Numeric variable (duration)	nr of consecutive days with ≥25°C	6'715	27'385	3.0 (1-10)	2003-2016
23	Numeric variable (duration)	nr of consecutive days with ≥26°C	3'049	12'119	2.9 (1-10)	2003, 2005-2016
24	Numeric variable (duration)	nr of consecutive days with ≥27°C	1'282	4'765	2.6 (1-10)	2003, 2005, 2006, 2012-2015

Tmean: Daily mean temperature; P: percentile

*Tmean reached the threshold temperature for at least one day
